# Pazopanib: a novel treatment option for aggressive fibromatosis

**DOI:** 10.1186/s13569-016-0061-3

**Published:** 2016-12-01

**Authors:** Gulcan Bulut, Anil Ozluk, Atike Pınar Erdogan, Ruchan Uslu, Nevra Elmas, Burcak Karaca

**Affiliations:** 1 Department of Medical Oncology, Ege University Medical School, Tulay Aktas Oncology Hospital, Ege University, Izmir, Turkey; 2 Department of Internal Medicine, Ege University Medical School, Ege University Medical School Hospital, Ege University, Izmir, Turkey; 3 Department of Radiology, Ege University Medical School, Ege University Medical School Hospital, Ege University, Izmir, Turkey

**Keywords:** Pazopanib, Aggressive fibromatosis, Desmoid tumor, Oral tyrosine kinase inhibitor

## Abstract

**Background:**

Aggressive fibromatosis (AF), also known as desmoid tumor, is an uncommon soft tissue neoplasm. AF does not metastasize, but it is locally invasive and its propensity for recurrence after conservative resection is well documented. No effective cytotoxic treatment has been reported, hence there is a need for novel treatment strategies.

**Case presentation:**

We present the case of an AF successfully treated with an oral tyrosine kinase inhibitor, pazopanib, with mild side effects. As far as we know, this is the first case of AF with complete response to pazopanib.

**Conclusion:**

Pazopanib might be an effective treatment option for AF.

## Background

Aggressive fibromatosis (AF) (also called deep fibromatosis or desmoid tumor) is a proliferation of cytologically benign-appearing fibrocytes, often resulting in significant functional loss. The nature of the lesion is controversial: some evidence suggests that it is a reactive process, whereas other evidence supports a neoplastic etiology [1]. Although it does not have the propensity of distant organ metastases, AF often exhibits an infiltrative pattern of spread in an abundant collagen matrix, giving it a dense, fibrotic character. As a result, this tumor can produce local tissue destruction leading to significant morbidity and functional loss.

Since the etiology of AF is poorly understood, several medical approaches have been combined with or without surgical resection with scarce results. These include chemotherapy with doxorubicin-based combinations, antiestrogen therapy with tamoxifen, nonsteroidal anti-inflammatory drugs (NSAIDs) such as indomethacin and sulindac, colchicines [2]. However, all of these treatment approaches show moderate activity. Due to a lack of efficacious treatment options, patients might die due to local organ dysfunction because of their locally progressive disease.

Sporadic AF is usually associated with somatic mutations in codons 41 or 45 of exon 3 of beta-catenin (CTNNB1). AF occurring in the background of familial adenomatous polyposis (FAP) usually contains inactivating germline mutations in the adenomatous polyposis coli (APC) gene. CTNNB1 and APC are part of the Wnt signaling pathway and mutations in either gene resulting in stabilization of the beta-catenin protein and allowing nuclear translocation and binding of beta-catenin to the T cell factor/lymphoid enhancer factor (TCF/Lef) family of transcription factors, lead to activation of target genes that may underlie desmoid tumor biology and clinical behavior. In the era of molecularly targeted therapeutics, there is a need to exploit the molecular mechanisms behind desmoid tumorigenesis and progression in a better way. Recently, new encouraging data with small molecule tyrosine kinase inhibitors (imatinib, sunitinib etc.,) have been published [3–7].

These new data support further investigation of the role of novel tyrosine kinases in AF. Pazopanib is one of the latest anti-angiogenic drugs developed to target VEGF and PDGF. It has recently been approved for the treatment of advanced renal cancer and soft-tissue sarcomas by the US Food and Drug Administration (FDA) and by the European Medicines Agency (EMA). [8, 9].

## Case report

Here we report a case of AF treated successfully with pazopanib. In 2013, a 50-year-old male was admitted to our University Hospital with recurrent episodes of abdominal pain, and loss of appetite. He did not have any comorbid disease and was not on any medication. Computed tomography (CT) scan showed an intra-abdominal soft tissue mass of 5 × 4 × 3 cm originating from the retroperitoneum. For both diagnostic and therapeutic reasons, he underwent a surgical excision of the mass, including partial resection of transverse colon. Pathological examination revealed aggressive fibromatosis with a low proliferation index. The surgical procedure was accepted as R2 resection of the residual mass.

In spite of the residual mass after surgery, and considering that the Ki-67 index was 3% and the patient was asymptomatic, no further treatment was offered at that time. A wait-and-see policy is one of the most acceptable options with slowly progressing AFs, since AF can show very varying clinical behavior, ranging from spontaneous regression to rapid progression leading to local organ dysfunction [10].

**Fig. 1:**
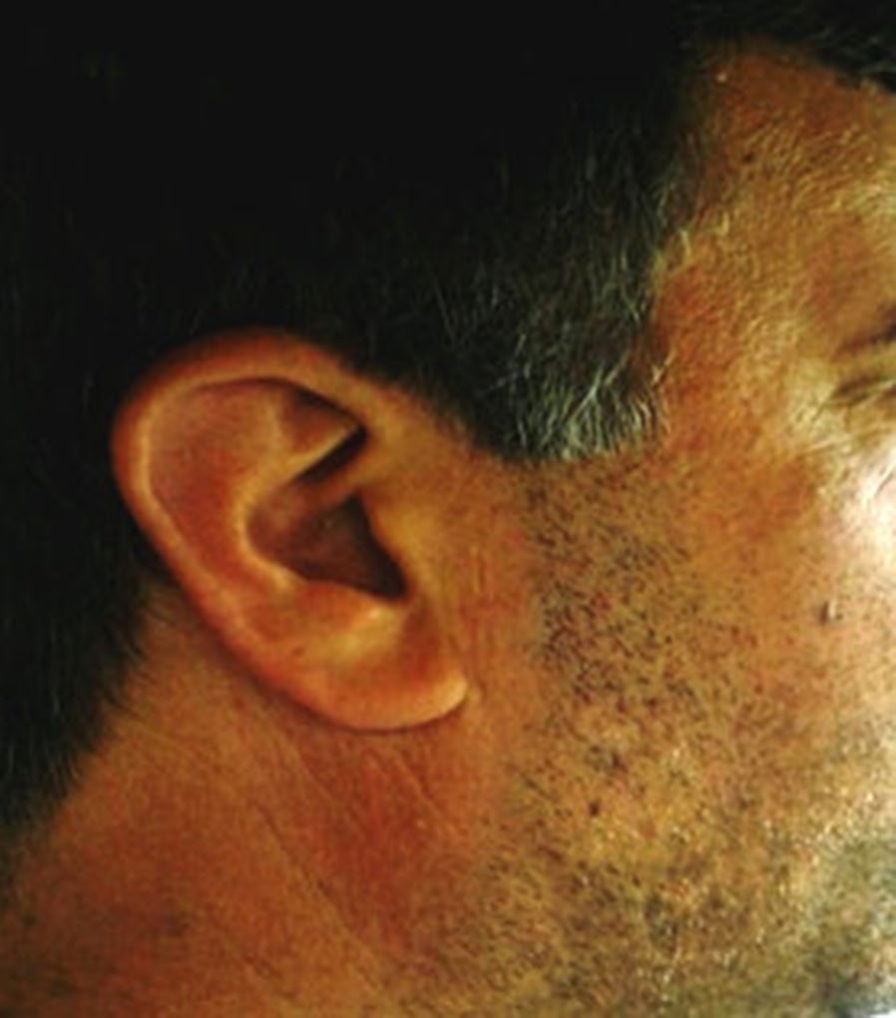
The photographs the patient before treatment pazopanib

**Fig. 2:**
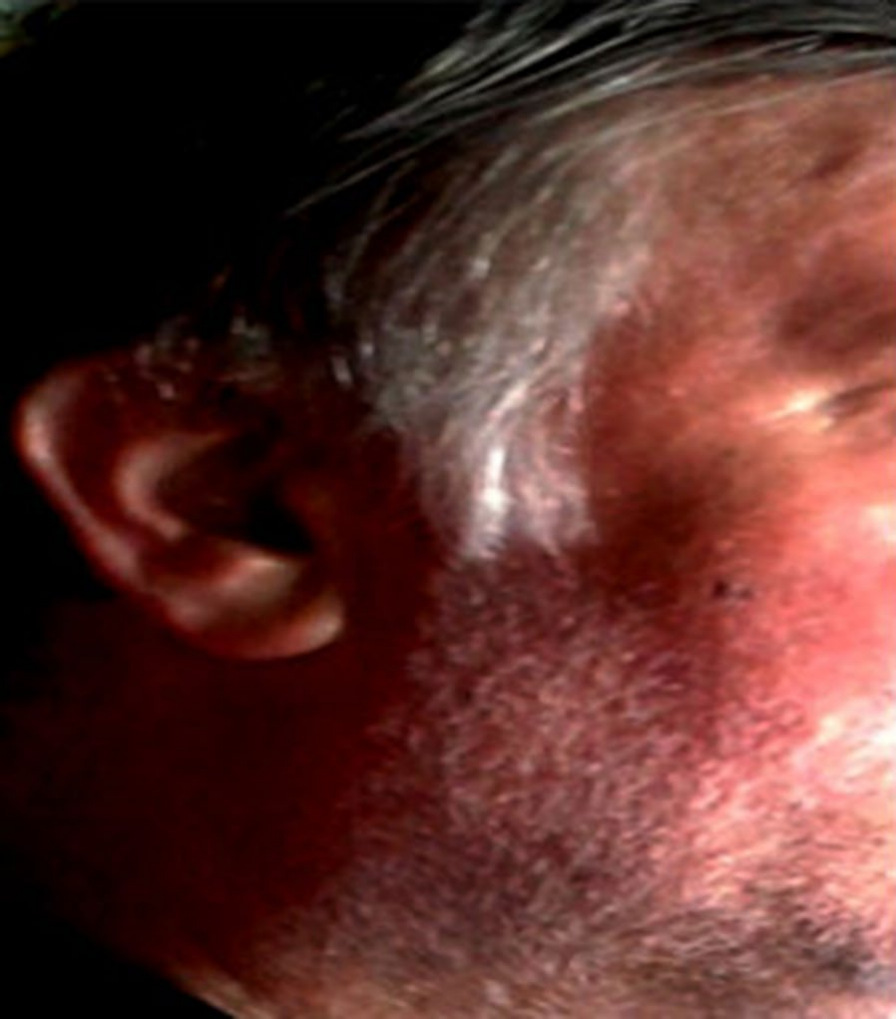
One month after the start of pazopanib treatment; Patient presented with loss of hair color and vitiligo

**Fig. 3:**
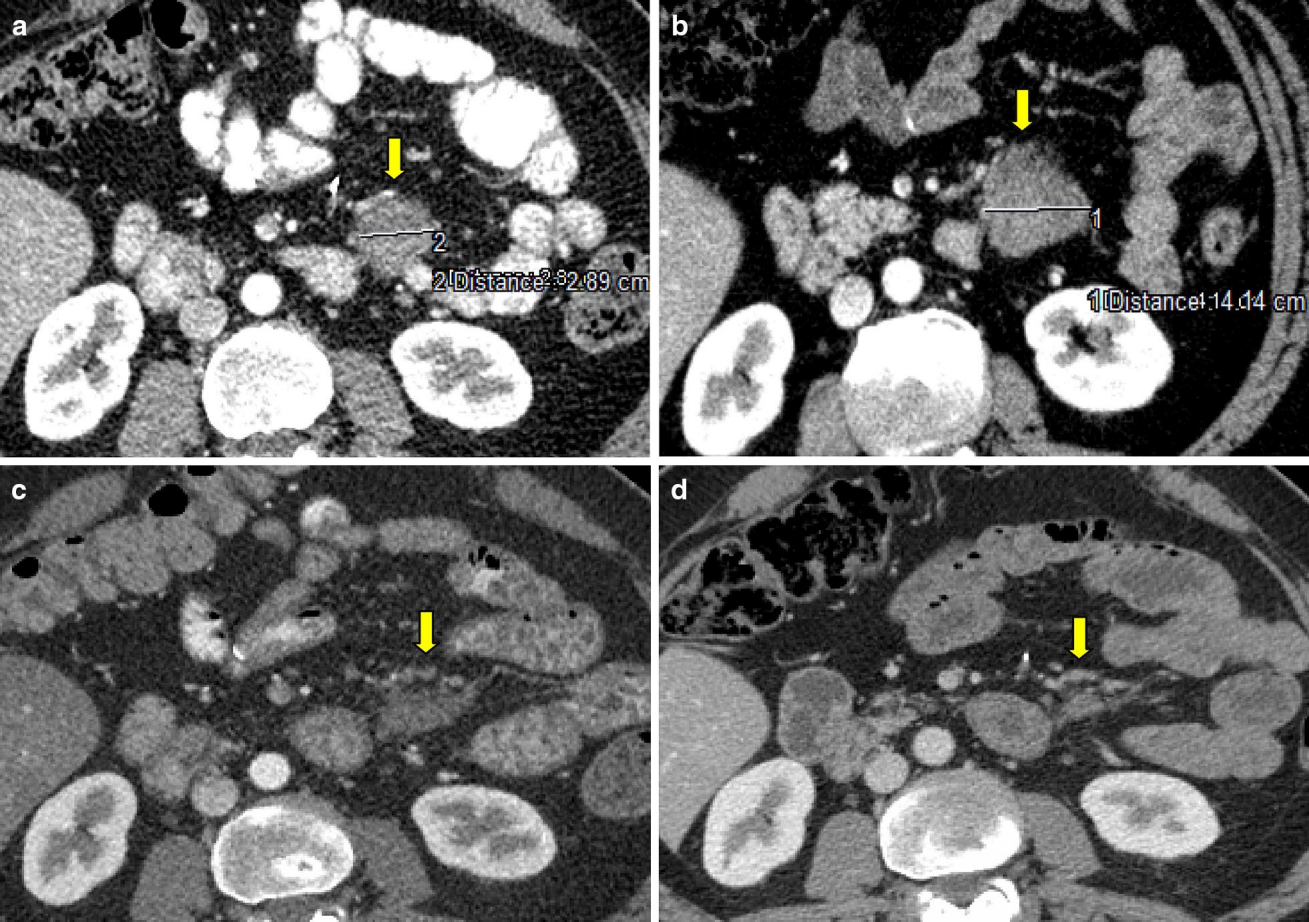
Patient CT scans; **a** before initial treatment of tamoxifen; axial plan CT scan performed in September 2013 demonstrates 2.89 cm diameter homogeneous mass located at the *left* anterior pararenal space of the retroperitoneal area (*yellow arrow*). **b** Post contrast CT images in december 2013; retroperitoneal mass (diameter 4.14 cm) progressed after treatment of tamoxifen (*yellow arrow*). **c** 9 months after the initiation of pazopanib treatment; the residual mass regressed on CT scan at same level (*yellow arrow*). **d** Post contrast CT images obtained in October 2015 showing complete response of the residual mass at 22 months of pazopanib treatment.(*yellow arrow*)

During his planned follow-up visits, CT scans revealed progression of residual mass and tamoxifen 20 mg/daily was started. After 3 months on tamoxifen, progressive disease was detected by CT scan (Fig. 3a, b). This time, the patient complained of abdominal cramps and his creatinine level was rising due to invasion of bladder by tumoral mass. Since no cytotoxic treatment was reported to have an efficacy for AF, we searched for possible treatment options on PubMed. Recently, new data has been released to demonstrate the efficacy of tyrosine kinases for AF [11]. It was suggested that kinase-targeting therapy may be effective against AF as there may be an autocrine/paracrine loop in AF that sustains platelet-derived growth factor receptor (PDGFR)-α and PDGFR-β activation [12]. Based on the literature that Pazopanib may be an effective treatment option in desmoid tumor/aggressive fibromatosis, we decided to treat our patient with pazopanib. The schedule was 800 mg/day. He tolerated the drug quite well with mild to moderate GI symptoms, including grade I diarrhea. One month after the initiation of pazopanib, he presented with vitiligo and hair depigmentation (Figs. 1, 2) which are quite common side effects of pazopanib.

On the follow-up period, first response to treatment was documented 4 months after the initiation of treatment (Fig. 3c). The response to treatment lasted on the follow-up visits, and after 22 months on pazopanib; complete response was achieved on patient’s last scan (Fig. 3d).

## Conclusion

Our report demonstrates that pazopanib is an effective and well-tolerated treatment option for the treatment of AF. To the best of our knowledge, this is the first reported case of AF where a complete response was achieved with pazopanib. AF has various clinical presentations, from spontaneous regression to rapid progression of tumor, necessitating a precise treatment decision. In our case, the patient was progressing not only radiologically but also clinically, which led us to decide in favor of a targeted treatment for this case.

Angiogenesis is one of the fundamental mechanisms in cancer and many studies suggest that it also plays a crucial role in soft tissue sarcomas [13]. Based on the results of a phase 3 randomized, placebo-controlled trial pazopanib was approved by the FDA in 2012 for the treatment of patients with locally advanced or metastatic soft tissue sarcoma after treatment with standard chemotherapy [14].

Owing to low or no cumulative toxicity of pazopanib compared to standard chemotherapy may allows an extended treatment duration. However, this observation clearly needs to be confirmed in prospective studies. The French Sarcoma Group has conducted a phase II trial that assesses the efficacy and toxicity of pazopanib in AF (ClinicalTrials.gov identifier NCT01876082). We hope that above mentioned clinical trial will confirm the effectiveness of pazopanib in AF, a challenging atypical tumor.

## Abbreviations

AF: aggressive fibromatosis
NSAIDs: nonsteroidal anti-inflammatory drugs
VEGF: vascular endothelial growth factor
PDGF: platelet derivated growth factor
FDA: US Food and Drug Administration
EMA: The European Medicines Agency
CT: computed tomography
PET: positron emission tomography
